# Supplementary material for: A deep learning framework for the localization of landmarks on the lateral semi circular canals
Source: PLoS One. 2026 May 12;21(5):e0348976. doi: 10.1371/journal.pone.0348976 (PMC13166903; doi:10.1371/journal.pone.0348976)
Supplement: Attachment — Submitted filename: Rebuttal.pdf [file pone.0348976.s002.pdf]

Dear Editor,

Thank you for giving us the opportunity to submit a revised version of our manuscript titled “A Deep Learning Framework for the Localization of Landmarks on the Lateral Semi Circular Canals” to PlosONE. We appreciate the time and effort that you and the reviewers have dedicated to providing feedback on our manuscript. We have modified the manuscript to reflect the suggestions provided. We have highlighted the changes within the manuscript. The manuscript has also been updated with the new LaTeX template available on the PlosONE website.

Here is a point-by-point response to the reviewers' comments and concerns. All the responses are in *italic* and the corresponding changes in the manuscript are referred to in this rebuttal letter using line numbers.

Academic Editor's comments

1. Please ensure that your manuscript meets PLOS ONE's style requirements, including those for file naming. The PLOS ONE style templates can be found here and here.
   We have ensured that the manuscript uses the PLOS ONE template and meets the style requirements.

2. We note that you have indicated that there are restrictions to data sharing for this study. For studies involving human research participant data or other sensitive data, we encourage authors to share de-identified or anonymized data. However, when data cannot be publicly shared for ethical reasons, we allow authors to make their data sets available upon request. For information on unacceptable data access restrictions, please see here.
   Before we proceed with your manuscript, please address the following prompts:
   a) If there are ethical or legal restrictions on sharing a de-identified data set, please explain them in detail (e.g., data contain potentially identifying or sensitive patient information, data are owned by a third-party organization, etc.) and who has imposed them (e.g., a Research Ethics Committee or Institutional Review

Board, etc.). Please also provide contact information for a data access committee, ethics committee, or other institutional body to which data requests may be sent. b) If there are no restrictions, please upload the minimal anonymized data set necessary to replicate your study findings to a stable, public repository and provide us with the relevant URLs, DOIs, or accession numbers. Please see http://www.bmj.com/content/340/bmj.c181.long for guidelines on how to de-identify and prepare clinical data for publication. For a list of recommended repositories, please see https://journals.plos.org/plosone/s/recommended-repositories. You also have the option of uploading the data as Supporting Information files, but we would recommend depositing data directly to a data repository if possible.

Please update your Data Availability statement in the submission form accordingly.

The data collected is owned by the Royal Victorian Eye and Ear Hospital, Melbourne, Australia. The ethics under which the data was collected by the University of Melbourne researchers restricts its public sharing. However, anonymised data can be shared on request. Please contact Prof Stephen O'Leary (sjoleary@unimelb.edu.au) the head of the Department of Otolaryngology in the University of Melbourne and also the owner of the ethics (#08/796H/18) of the data. Researchers can also contact Kerryn Baker (kerryn.baker@eyeandear.org.au), Education Precinct Manager of the Royal Victorian Eye and Ear Hospital, about the ethics.

3. We note you have included a table to which you do not refer in the text of your manuscript. Please ensure that you refer to Table 4 in your text; if accepted, production will need this reference to link the reader to the Table.
   Thank you for noticing this oversight. We have corrected this.

4. If the reviewer comments include a recommendation to cite specific previously published works, please review and evaluate these publications to determine

whether they are relevant and should be cited. There is no requirement to cite these works unless the editor has indicated otherwise.

We feel that the reviewers' comments are very constructive and neither has requested us to cite references that are not relevant.

Additional Editor Comments:

Reviewer 1 provides very helpful advice regarding additional statistical information, tests, and parameters that should be reported. I further agree that, most importantly, the authors need to include an external validation dataset, as the original dataset contains only n =20 patients and was further subdivided for training and testing.

We admit that n=20 is small. We have however taken steps (such as augmentation and cross validation) to address this. Also, please note that Reference #21 of the paper in the field have used similar (or lesser) number of patients. However, we have done further validation on 14 new patients. Please note that due to practical considerations such as data collection, labelling by experts and time constraints for the first author who is a PhD student in his final year, we have limited the external validation to 14 patients. Please note that we have called this 'additional validation' as the dataset was drawn from the same population as the training and test sets. The changes to the manuscript are in lines 296-316.

We agree that the additional statistical tests suggested will add value and have conducted them (lines 305–315, figures 13, 14 tables 4).

Reviewer 1's comments

1. Sample Size and Generalizability: The dataset consists of only 20 patients, all with normal anatomy. Even with augmentation, this raises concerns about robustness and generalizability. Inclusion of a larger and more heterogeneous dataset, ideally with pathological cases, would significantly strengthen the study.

Thank you for the comments. We agree with the reviewer. Unfortunately, we have been limited in the number of patients due to practical issues in collecting and labelling data. We have taken measures such as augmentation to overcome issues of over-fitting and cross validation.

However, as per your suggestion, we have further conducted an external validation that addresses the issue of generalizability. Please note that we have called this 'additional validation' as the dataset was drawn from the same population as the training and test sets. Please see Section Additional Validation of the manuscript.

This patient cohort for this study were cochlear implant patients. As such, it is rare for any abnormalities and pathologies in and around the lateral semi-circular canals (where the landmarks are located) to be present. We plan to extend the method to include pathological cases in the future. This has been added as a limitation in the discussion (lines 342-345).

2.External Validation: The absence of an external validation cohort is a major limitation. Testing on independent datasets is essential to demonstrate clinical applicability.

Thank you. We acknowledge this is an issue and have conducted an additional validation (Section Additional Validation, Table 4 etc).

3.Statistical Rigor: While mean and standard deviation values are reported, the statistical analysis could be improved. Confidence intervals, hypothesis testing, or Bland–Altman plots would better substantiate the claim of equivalence to human experts.

Thank you for this suggestion. We have conducted these statistical analyses. Lines 305-315 and table 4, figures 13, 14 in the manuscript reflect these changes. Please note that we have used non-parametric statistics for this analysis as the number of

patients in the additional validation set was 14 with no augmentations. This leads to the violation of the normality assumption required to conduct parametrics statistics.

4.ROI Determination: The exhaustive crop-size approach is functional but inefficient. Alternative approaches (e.g., attention mechanisms, transformer-based localization, or adaptive ROI detection) could be considered to modernize the framework.

We agree that this approach of crop size detection is not the most optimal. We will conduct more efficient crop size detection methods such as those suggested by the reviewer in future work (lines 356-357).

5.Interpretability and Clinical Workflow: The manuscript would benefit from visualization of errors, attention maps, or examples of both successes and failures. Furthermore, details such as runtime per scan and workflow integration into surgical planning should be elaborated.

We have added figures to visualise results including errors (Figures 15).

We did not include run time per scan as it is a subjective measure depending on factors such as processing power and memory capacity of the computer.

Automating the identification of landmarks enables patient scans to be transformed into a standardized coordinate system, improving the clinical workflow by (1) facilitating automation of downstream tasks such as multi-modal registration, surgical planning, and pathology identification, and (2) minimizing the need for human intervention. This has been added to the manuscript (lines 318-322).

6.Data Availability: The dataset is not publicly available due to ethical restrictions, which limits reproducibility. While this is understandable, stronger justification or provision of anonymized subsets would help align with open science practices.

The data collected is owned by the Royal Victorian Eye and Ear Hospital, Melbourne, Australia. The ethics under which the data was collected by the

University of Melbourne researchers restricts its public sharing. However, anonymised data can be shared on request. Please contact Prof Stephen O'Leary (sjoleary@unimelb.edu.au) the head of the Department of Otolaryngology in the University of Melbourne and also the owner of the ethics (#08/796H/18) of the data. Researchers can also contact Kerryn Baker (kerryn.baker@eyeandear.org.au), Education Precinct Manager of the Royal Victorian Eye and Ear Hospital, about the ethics.

Reviewer 2's comments

1. According to the data shown in "[8]:Copson B, Wijewickrema S, Slinger C, Youssef D, Gerard JM, O'Leary S. Definition of a coordinate system for multi-modal images of the temporal bone and inner ear. Plos one. 2024", intra and inter rater (experts) variation is very low. This means that it is an easy and consistent task for the "experts". How do the authors see the value of this system?

Automating the identification of landmarks enables patient scans to be transformed into a standardized coordinate system, improving the clinical workflow by (1) facilitating automation of downstream tasks such as multi-modal registration, surgical planning, and pathology identification, and (2) minimizing the need for human intervention. This has been added to the manuscript (lines 318-322).
