# Supplementary material for: A deep learning framework for the localization of landmarks on the lateral semi circular canals
Source: PLoS One. 2026 May 12;21(5):e0348976. doi: 10.1371/journal.pone.0348976 (PMC13166903; doi:10.1371/journal.pone.0348976)
Supplement: Attachment — Submitted filename: Rebuttal_auresp_2.pdf [file pone.0348976.s003.pdf]

Dear Editor,

Thank you for giving us the opportunity to submit a revised version of our manuscript titled “A Deep Learning Framework for the Localization of Landmarks on the Lateral Semi Circular Canals” to PlosONE. We appreciate the time and effort that you and the reviewers have dedicated to providing feedback on our manuscript. We have modified the manuscript to reflect the suggestions provided. We have highlighted the changes within the manuscript. The manuscript has also been updated with the new LaTeX template available on the PlosONE website.

Here is a point-by-point response to the reviewers’ comments and concerns. All the responses are in *italic* and the corresponding changes in the manuscript are referred to in this rebuttal letter using line numbers.

Journal Requirements:

1. If the reviewer comments include a recommendation to cite specific previously published works, please review and evaluate these publications to determine whether they are relevant and should be cited. There is no requirement to cite these works unless the editor has indicated otherwise.

Not relevant

2. Please review your reference list to ensure that it is complete and correct. If you have cited papers that have been retracted, please include the rationale for doing so in the manuscript text, or remove these references and replace them with relevant current references. Any changes to the reference list should be mentioned in the rebuttal letter that accompanies your revised manuscript. If you need to cite a retracted article, indicate the article’s retracted status in the References list and also include a citation and full reference for the retraction notice.

Thank you for the reviewer’s point, we have added a new reference to elaborate why we chose 0.5mm as the upper boundary for the statistical comparison. The new reference is: Schneider D, Hermann J, Mueller F, Braga GO'TB, Anschuetz L,

Caversaccio M, Nolte L, Weber S, Klenzner T. Evolution and Stagnation of Image Guidance for Surgery in the Lateral Skull: A Systematic Review 1989–2020. Front Surg. 2021;7:604362. (line 308)

Additional Editor Comments:

I would note that in Fig. 15 it is quite difficult to see the landmarks that are being shown. I would suggest making the dot that designates the landmark(s) bigger. I also suggest making this figure colorblind friendly (e.g. red and blue instead of red and green).

Thank you for pointing this out. We have updated Fig 15 using bigger landmarks with the colors red and blue. (Fig 15)

Reviewer 3's comments

Thank you for the opportunity to review the manuscript entitled "A Deep Learning Framework for the Localization of Landmarks on the Lateral Semicircular Canals." This study presents an innovative application of deep learning to a longstanding challenge in otologic and vestibular research. Automated and reproducible localization of canal landmarks has potential to advance both clinical and morphometric investigations of the inner ear.

Visualizing the detailed morphology of the endosseous and membranous labyrinths remain one of the biggest challenges in ontological research. In this context, the authors' effort to develop a computational framework for landmark detection is both appropriate and potentially impactful.

While the deep learning architecture and training procedures fall outside my primary area of expertise, I am happy to provide some feedback on the morphometric study

of the LSCC. Below, I offer several suggestions focused on the morphometric and anatomical aspects of the study.

• In your “Landmarks of the LSCC” section, you need a clearer definition of where each landmark was placed in reference to anatomical features (e.g., “middle of the bony ampulla and point at which the slender LSCC joins the vestibule”). Please add in this description of the features.

Thank you for your comment. We have added a clearer definition in the section “Landmarks of the LSCC”. The Anterior Landmark was defined as the centre-point of the junction between the anterior aspect of the LSCC, and the utricle - that is, the point at which the slender bony canal opens into the vestibule anteriorly - confirmed in all three imaging planes. The Posterior Landmark was defined equivalently at the posterior junction of the LSCC and the utricle, where the canal re-enters the vestibule posteriorly, again confirmed in all three planes. (line 104 to 109)

• Line 215: “Fi” should be “Fig”.

Fixed, thank you. (line 220)

• Line 298: What is meant by the word “annotated”?

Thank you for asking, it is a term used in deep learning which represents the process used to label the ground truth. For example, in our automatic landmarks detection task, it means the ground truth landmarks of the 14 additional patients’ scans were manually identified by expert clinicians. (No change in the manuscript)

• Lines 300 and 313: You say the upper boundary for the clinically significant distance threshold is 0.5mm and cite Mueller et al., 2021. That paper reported ~0.5mm as the maximum distance found between the facial recess trajectory orientation to the facial nerve and osseous spiral lamina. However, the average distances they report are 0.44mm, and 0.35mm, respectively. I would recommend elaborating here on why exactly 0.5mm is the best fit for establishing your upper boundary?

Thank you for pointing this out. We agree that the literature Mueller et al., 2021 is insufficient for the choice of the upper boundary 0.5mm. We have cited another paper Schneider et. al., which clearly suggested it as the threshold for lateral skull based image guidance. The elaboration has been added in the manuscript: Accuracy requirements in image guided surgery and image registration using landmarks are not standardised, and reasonable thresholds depend on the precision required by the surgical technique. For lateral skull base image guidance, a value of 0.5mm has been suggested (Schneider et. al.), and we adopted this as the predefined upper boundary for evaluating the clinical acceptability of our landmark detection results. (line 305 to 309; line 321; line 350)

The reference added: Schneider D, Hermann J, Mueller F, Braga GO'TB, Anschuetz L, Caversaccio M, Nolte L, Weber S, Klenzner T. Evolution and Stagnation of Image Guidance for Surgery in the Lateral Skull: A Systematic Review 1989–2020. Front Surg. 2021;7:604362.

• Line 331: What is meant by “ground truth”?

Thank you for asking, “ground truth” is a term used in supervised deep learning which represents the target of the model’s optimization direction. In our task it is the median of the landmarks coordinates picked by the experts. The model’s training process is the optimization to minimize the distance between the predicated landmarks and the ground truth landmarks. (No change in the manuscript)

• Line 332: “…coordinates selected by human experts (citation needed)”

Thanks for the comment. We have added the citation. (line 336)

• Figure 13. It seems the greater variability in the posterior landmark is driven by one individual. Can you elaborate on why this may be? It might be worth doubling checking the scan to see what is going on here.

Thank you for pointing this out. We have checked the scans of the greatest difference shown in Figure 13’s posterior landmarks. The scans are not from a single patient, they come from three different patients. As far as we can see, there is no obvious pattern in these three cases. We have also observed the slightly higher

variation in the posterior landmark coordinates. This is likely because of higher variations in the human landmark detections which were used as ground truth for training our models. We have already addressed this in the discussion. The reason behind the differences in human landmarks detections for anterior and posterior points may be the higher anatomical complexity around posterior points, as when manually annotated, the point where the lateral semicircular canal enters the vestibule is on an oblique plane. In addition, there is variation in the anatomic location of the confluence with the posterior semicircular canal, which can make this landmark susceptible to inter-observer variability. We have added this to the discussion for clarification (line 340 to 345).
